# Phacoemulsification in patients with uveitis: long-term outcomes

**DOI:** 10.1186/s12886-020-01373-5

**Published:** 2020-03-17

**Authors:** Serdar Ozates, Nilufer Berker, Pinar Cakar Ozdal, Yasemin Ozdamar Erol

**Affiliations:** 1 Department of Ophthalmology, Kars Harakani State Hospital, Yenişehir Mahallesi, İsmail Aytemiz Blv. No:55, 36200 Merkez, Kars Turkey; 2 Department of Ophthalmology, Ulucanlar Eye Training and Research Hospital, Ankara, Turkey

**Keywords:** Phacoemulsification, Uveitis, Cystoid macular edema, Posterior capsule opacification, Glaucoma

## Abstract

**Background:**

To assess the long-term outcomes of phacoemulsification and intraocular lens (IOL) implantation in eyes with uveitis.

**Methods:**

One hundred and five eyes of 81 patients, who underwent phacoemulsification and IOL implantation between January 2009 and July 2016, were included in this study. The demographic data, preoperative clinical findings, postoperative outcomes, and intraoperative and postoperative complications were recorded. All collected data and risk factors with regard to visual prognosis were analyzed with the help of the Statistical Package for the Social Sciences version 20.0 software program (IBM Corp., Armonk, NY, USA).

**Results:**

During follow-up (mean: 35.2 ± 22.2 months), corrected distance visual acuity (CDVA) improved in 87.7% of all eyes and reached a level of 0.3 LogMAR or greater in 61.3% of eyes. Postoperative complications included posterior capsule opacification (50.9%), posterior synechiae (21.7%), cystoid macular edema (16%), epiretinal membrane (13.2%), glaucoma (11.3%), increased intraocular pressure (8.5%), and severe inflammation (6.6%). Uveitis recurred in 55.7% of all eyes. The risk for the development of cystoid macular edema was found to be associated with recurrence in the early postoperative period. Low visual acuity risk was 11.1-fold higher with macular scarring (*p* = 0.001) and 14-fold higher with optic atrophy (*p* < 0.001), respectively.

**Conclusions:**

With appropriate management during the pre- and postoperative periods, phacoemulsification and IOL implantation surgery can be safe and effective in eyes with uveitis. However, great caution must be taken to prevent complications both before and after the surgery.

## Background

Uveitis is a common eye disorder and causes anatomic and functional damage and complications in anterior and posterior segment structures due to underlying inflammatory processes [1, 2]. Therefore, the prevention and treatment of this structural damage become crucial. One of the most frequent complications that occur in patients with uveitis is cataract formation. Cataract may arise due to the inflammation itself or as a result of corticosteroid treatments [3].

Successful cataract management has been reported in several previous studies that have documented favorable anatomical and functional success rates with phacoemulsification and intraocular lens (IOL) implantation [4–7]. However, cataract surgery in eyes with uveitis poses a number of intraoperative and postoperative challenges, such as miotic pupil, synechiae, anterior segment bleeding, iris atrophy, excessive inflammation, high intraocular pressure (IOP), and cystoid macular edema (CME) [5, 8, 9]. The complication rate after phacoemulsification and IOL implantation can be reduced in the short term by careful preoperative medication, competent surgery, and close postoperative follow-up. However, the long-term effects of this surgical procedure may vary under different circumstances. Long term outcomes of phacoemulsification and IOL implantation in eyes with uveitis have shown great variations in previously reported studies, with variations arising due to differences in etiologic factors, phacoemulsification systems, surgical experience, and IOL type [10].

Cataract surgery may lead to serious complications in eyes with chronic inflammation [9, 11]. Previous studies have reported that eyes with uveitis often have unfavorable surgical results due to low tolerance to surgery and IOL implantation; however, the success rates of surgery have increased today as a result of recent developments in surgical techniques and IOL materials [12, 13].

The aim of this study is to investigate the long-term outcomes of cataract surgery and the visual outcomes, complications, and contributing factors in patients with uveitis.

## Methods

This retrospective study was conducted at Ulucanlar Eye Training and Research Hospital, Ankara, Turkey, in accordance with the ethical standards of the Declaration of Helsinki. The study protocol was approved by the institutional review board of Ankara Numune Training and Research Hospital.

Patients who met the eligibility criteria and who underwent phacoemulsification and IOL implantation between January 2009 and July 2016 were included in the study. Exclusion criteria included a history of previous ocular surgery, and the development of new ocular disease without an association with uveitis. Patients with systemic disease–associated uveitis, such as Behçet disease and seronegative spondyloarthropathy, were included in the study; however, patients with systemic diseases that may have ocular effects other than uveitis, such as diabetes mellitus, were excluded from the study. Patients with active uveitis or a remission period of less than 3 months were also excluded. Patients with seronegative spondyloarthropathy formed the rheumatic disease associated uveitis group.

In order to prevent the exacerbation of uveitis following surgery, all patients other than those with presumed herpetic uveitis and Fuchs uveitis syndrome (FUS) received oral *prophylactic* corticosteroid 0.5 mg/kg/day for 2 weeks before the surgery [11]. Patients with presumed herpetic uveitis received oral acyclovir 800 mg/day for 1 month before the surgery, even if patients were in remission [13, 14]. No changes were made in immunosuppressive treatment protocols of the patients. All phacoemulsification and IOL implantation procedures were performed by the same surgeon (N. B.). Iris retractor were used in patients with poorly dilated pupil if needed. After the surgery, all patients received topical moxifloxacin 0.5% six times a day for 2 weeks and dexamethasone 0.1% every hour for 1 week. Topical and oral corticosteroid treatments were tapered according to the patient’s postoperative inflammation level. Patients with presumed herpetic uveitis received oral acyclovir 800 mg/day for 1 month after the surgery [13, 14]. Topical ketorolac tromethamine 0.5% was administered to patients with posterior capsule rupture or a previous history of CME. Patients with postoperative IO*P* values over 21 mmHg received topical beta-blockers, alpha-2 agonists or carbonic anhydrase inhibitors based on the clinical approach.

All patients underwent a complete ophthalmological examination at every postsurgical control visit. Total refractive error was measured with an auto refractometer (Topcon KR-880 Auto Kerato-Refractometer, Topcon, Japan). The corrected distance visual acuity (CDVA) was determined using a Snellen chart and all CDVA data were converted into logarithm minimal angle resolution (logMAR) for statistical analysis. Anterior chamber reaction was evaluated according to the Standardization of Uveitis Nomenclature classification, and vitreous haze was evaluated according to the Nussenblatt vitreous haze classification [15, 16]. A vitreous haze grade of 2 or higher that caused a decrease in CDVA was considered as vitreous opacification. A postoperative inflammation level of three or higher in the anterior chamber was considered to constitute severe postoperative inflammation. Recurrence rate corresponds to number of recurrences per year during the follow-up period. The CME was diagnosed based on fundus examination, fundus florescein angiography, and optical coherence tomography.

Statistical analyses were performed using the Statistical Package for the Social Sciences Statistics version 22.0 software program (IBM Corp., Armonk, NY, USA). The Shapiro–Wilks W test was used to evaluate the normal distribution of the data. The outcomes were reported as mean value and standard deviation. The independent samples t-test was used to determine differences in outcomes such between two independent groups, while the analysis of variance was used for comparisons of three or more groups. Paired samples t-test was used to determine differences in pre- and postoperative levels of outcomes such as CDVA. Pearson’s correlation analysis performed to reveal the relation between quantitative and continuous variables. Chi-square test was used to determine differences in categorical variables between the groups. Relative risk (RR) with 95% confidence interval (CI) was calculated to reveal the risk factors. The statistically significant level was assumed to be *p* < 0.05 for all tests. Bonferroni correction was used in multiple comparisons.

## Results

The present study included 105 eyes of 81 patients. Eight eyes of eight patients were lost to follow-up prematurely. Of these eight patients, etiologic cause was FUS in five and idiopathic anterior uveitis in three, respectively. Table 1 presents the demographic data and characteristics of the patients and the distribution of uveitis. No significant differences were found between uveitis types in terms of age and gender (*p* = 0.373 and *p* = 0.323, respectively). Table 2 shows the sub-group analysis based on uveitis etiology.

**Table 1:** Characteristics of the patients, etiological causes and classification of uveitis

***Population characteristics***	***Value***
Number of patients and eyes	81/105
Male / Female	45/36
Mean age (years) *(Mean*)	51.2 ± 15.2 (min:19, max:87)
Mean follow-up period (months) *(Mean*)	50.8 ± 40.0 (min:13, max:188)
Mean postoperative follow-up period (months) *(Mean*)	35.2 ± 22.2 (min:10, max:120)
Mean preoperative remission period (months) *(Mean*)	13.3 ± 8.6 (min:4, max:59)
***Etiology***	
Fuchs uveitis syndrome (eyes)	26 (24.8%)
Behçet uveitis (eyes)	26 (24.8%)
Presumed herpetic uveitis (eyes)	12 (11.4%)
Idiopathic uveitis (eyes)	34 (32.4%)
Rheumatic disease associated uveitis (eyes)	7 (6.6%)
***Anatomic classification***	
Anterior uveitis (eyes)	62 (59%)
Intermediate uveitis (eyes)	7 (6.7%)
Posterior uveitis (eyes)	9 (8.7%)
Panuveitis (eyes)	27 (25.7%)

**Table 2:** Sub-group analysis based on uveitis etiology

***P*Value**				
	*FUS*	*BU*	*IU*	*RDAU*
**Postoperative visual acuity** (at 12th month)				
*PHU*	0.259	0.436	0.263	0.952
*FUS*		< 0.001^a^	0.123	0.785
*BU*			0.015^a^	0.852
*IU*				0.403
**Postoperative inflammation**				
*PHU*	0.738	0.293	0.738	0.492
*FUS*		0.025^a^	0.380	0.297
*BU*			0.128	0.308
*IU*				0.605
**Recurrence**				
*PHU*	0.738	0.293	0.738	0.492
*FUS*		0.016^a^	0.380	0.297
*BU*			0.128	0.308
*IU*				0.605
**Posterior Capsule Opacification**				
*PHU*	0.071	0.004^a^	0.723	0.102
*FUS*		0.269	0.198	0.521
*BU*			0.144	0.939
*IU*				0.141
**Cystoid Macular Edema**				
*PHU*	0.999	0.011^a^	0.344	0.105
*FUS*		< 0.001^a^	0.222	0.699
*BU*			0.015^a^	0.383
*IU*				0.274
**Glaucoma**				
*PHU*	0.251	0.818	0.144	0.285
*FUS*		0.830	0.731	0.132
*BU*			0.286	0.084
*IU*				0.184

*PHU* presumed herpetic uveitis, *FUS* Fuchs uveitis syndrome, *BU* Behçet uveitis, *IU* Idiopathic uveitis, *RDAU* rheumatic disease associated uveitis, ^a^ Statistically significant

During phacoemulsification, 71 eyes were implanted with one-piece acrylic hydrophobic IOLs, 33 eyes were implanted with one-piece acrylic hydrophilic IOLs, and one eye was implanted with a three-piece acrylic hydrophobic IOL. One eye additionally was implanted with a scleral fixated IOL after the cataract surgery.

### Visual acuity

**Fig. 1:**
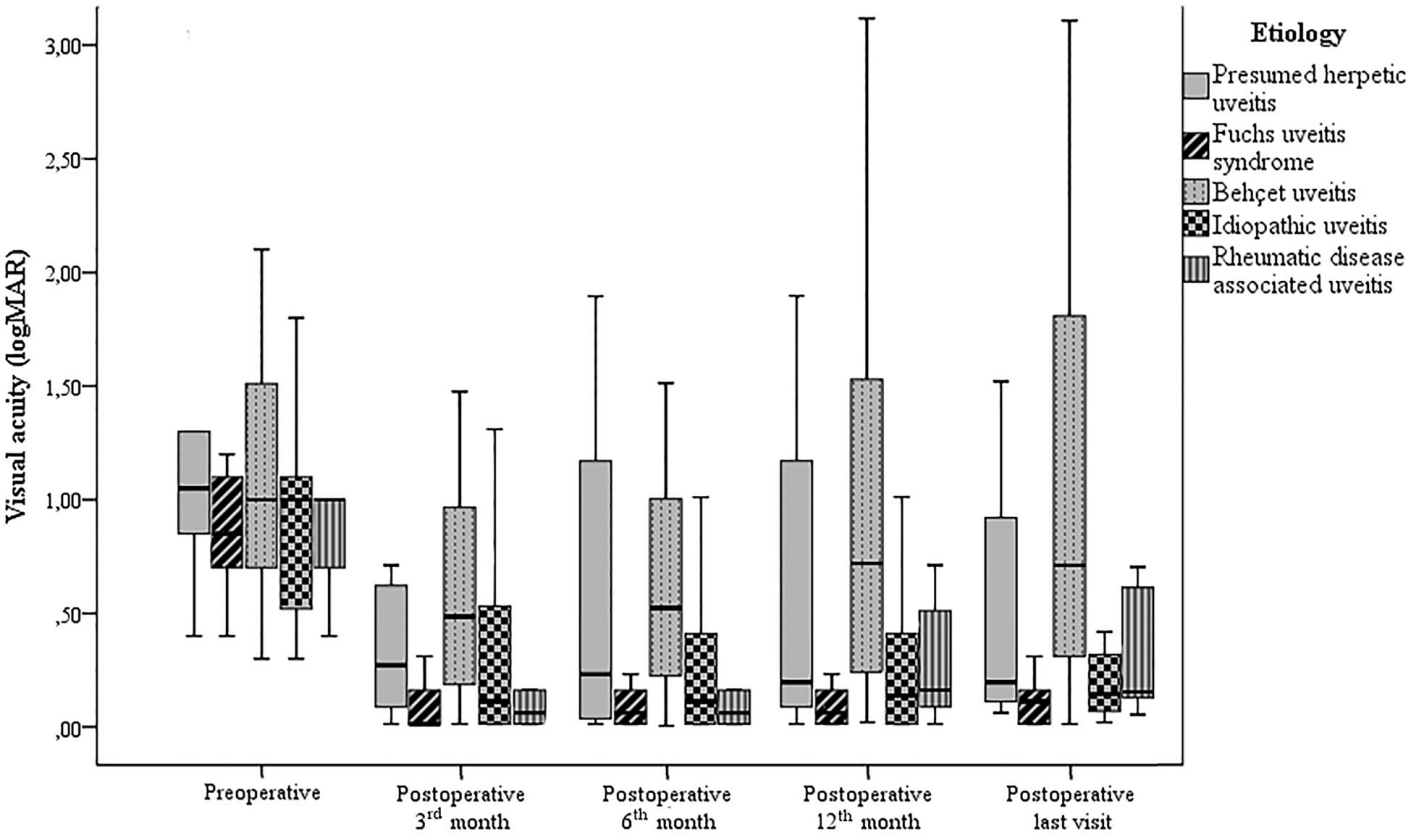
The preoperative and postoperative mean visual acuity values

Figure 1 shows the preoperative and postoperative mean CDVA values for different etiologic causes. The mean postoperative CDVA value at all control visits was significantly better than the mean preoperative CDVA value (*p* < 0.001 for all). The mean postoperative CDVA value after the first postoperative week was significantly better than CDVA at postoperative the first day (*p* < 0.001 for all) and did not significantly change throughout the follow-up (*p* > 0.05 for all). At the end of the follow-up, CDVA gain was achieved in 80.2% of the eyes and 61% of eyes reached a CDVA of 0.3 logMAR or better. Additionally, the mean CDVA value was better in eyes with FUS than in those with Behçet uveitis (BU) during the whole follow-up period (*p* < 0.001) and better in eyes with idiopathic uveitis than in those with BU after the first month postoperatively (*p* < 0.001). The mean CDVA value was significantly better in eyes with anterior uveitis than in eyes with panuveitis during follow-up (*p* < 0.001), whereas it was better than in eyes with posterior uveitis after the first month postoperatively (*p* < 0.05). Statistical analysis in our study revealed that the risk of low visual acuity after phacoemulsification and IOL implantation was 11.1-fold higher in eyes with macular scarring (95% CI: 1.9–63.5; *p* = 0.001) and 14-fold higher in eyes with optic atrophy (95% CI: 4.0–48.5; *p* < 0.001), respectively.

### Postoperative inflammation

No significant difference was noted between the etiological causes in terms of the postoperative anterior chamber reaction (*p* = 0.821). Of note, surgical manipulations, such as releasing synechiae, anterior vitrectomy, and the implantation of capsule tension ring, had no statistically significant effect on the anterior chamber reaction (*p* > 0.05 for all); however, iris hook insertion significantly induced the postoperative anterior chamber reaction (*p* < 0.001). Release of synechia was performed in 37 eyes and no significant relation was observed between the release of synechiae and postoperative inflammation (*p* > 0.05). In eyes with FUS, significantly lower anterior chamber reaction was present in those with hypochromia versus without (*p* = 0.027). Although, statistically insignificant, hydrophobic acrylic IOLs were well-tolerated and induced less postoperative inflammation in comparison with hydrophilic IOLs (*p* = 0.164).

### Recurrence

The mean preoperative recurrence rate was 3.0 ± 2.3 per eye and the mean preoperative remission period was 13.3 ± 8.65 months. The mean postoperative recurrence rate was 1.2 ± 1.9 per eye and the mean period until the first postoperative recurrence was 6.1 ± 4.9 months. The mean postoperative recurrence risk was 2.3-fold higher in eyes with a preoperative remission period of less than 1 year (RR: 2.31, 95% CI: 1.04–5.04; *p* = 0.03).

### Posterior capsule Opacification

In this study, 50.9% of all eyes developed posterior capsule opacification (PCO) during follow-up. The mean period between cataract surgery and the development of PCO was 14.9 ± 17.2 months. The development of PCO was significantly more frequent in eyes with FUS, BU, and rheumatological uveitis as compared with in eyes with presumed herpetic uveitis (*p* = 0.007, *p* < 0.001, and *p* = 0.013, respectively). No significant difference was observed between granulomatous and nongranulomatous uveitis types with respect to PCO development (*p* = 0.343). The development of PCO was significantly less frequent in eyes implanted with one-piece hydrophobic acrylic IOLs than in those with one-piece hydrophilic acrylic IOLs (*p* = 0.013). No statistically significant relation was found between the development of PCO and the preoperative recurrence number, mean remission period, presence of posterior synechiae, or postoperative anterior chamber reaction (*p* > 0.05 for each). A moderate and positive correlation was found between the period until the first postoperative recurrence and the period until PCO development (r = 0.480, *p* = 0.002). The eyes with earlier recurrence showed earlier and more pronounced PCO development versus postoperatively quiescent eyes.

### Cystoid macular edema

The mean period until the development of CME was 7.0 ± 3.8 months. CME developed more frequently in eyes with BU than in those with other etiologies (*p* < 0.05 for each). The number of recurrences was significantly higher in eyes with CME than without (*p* < 0.001). A strong and positive correlation was found between the postoperative period until the development of CME and the period until the first postoperative recurrence. Eyes with earlier postoperative recurrence showed earlier CME development as compared with postoperatively quiescent eyes (r = 0.827, *p* < 0.001). Eyes with a preoperative CME history developed significantly more CME after phacoemulsification (*p* < 0.001). In these eyes, the risk for CME development after phacoemulsification was 6.7-fold higher than in those with no preoperative CME history (RR: 6.7, 95% CI: 3.5–12.7).

### Intraocular pressure and Glaucoma

In total, 12 eyes developed glaucoma during follow-up, and two underwent trabeculectomy due to medically uncontrolled increase in IOP. The etiologic causes were FUS in two patients that underwent trabeculectomy. No significant relation was observed between glaucoma and the etiologic causes (*p* > 0.05 for each). There was a statistically significant higher risk of glaucoma development in eyes with higher postoperative IOP values (*p* = 0.004). Our study revealed that the glaucoma development risk was 5.4-fold higher in eyes with postoperative high IOP that required medical antiglaucomatous treatment (RR: 5.4, 95% CI: 2.1–13.8).

## Discussion

### Visual acuity

Various outcomes have been reported in previous reports regarding CDVA after phacoemulsification and IOL implantation in patients with uveitis [4, 9, 17]. Kosker et al. reported a CDVA value of 0.3 logMAR or better in 94.5% of eyes at the end of a follow-up period of 6 months [17]. Estafanous et al. noted that CDVA increased in 95% of eyes at the end of a follow-up period of 20 months, and that CME, glaucoma, and optic atrophy were the main reasons for low CDVA [9]. In our study, CDVA improved one line or more in 87.7% of eyes at the first month postoperatively. At the end of our follow-up period of a mean of 35.2 ± 22.2 months, the CDVA value was 0.3 logMAR or better in 61.3% of the eyes. Our results also revealed that the low visual acuity risk after phacoemulsification and IOL implantation was 11.1-fold higher in eyes with macular scarring and 14-fold higher in eyes with optic atrophy. Similarly, Elgohary et al. and Ram et al. reported macular scarring and optic atrophy as independent risk factors for low visual acuity after phacoemulsification [4, 18]. ERM and CME were also recognized as factors associated with a poor prognosis regarding postoperative visual acuity [19]. The lower CDVA outcomes in our study were considered to be associated with the longer follow-up period as compared with similar reports as well as the referral pattern of our specialized eye hospital, and high frequency of BU causing irreversible retinal damage.

### Postoperative inflammation

Despite intensive preoperative prophylaxis, severe postoperative inflammation that required additional treatment was observed in 6.6% of all eyes in our study. In the literature, severe postoperative inflammation was reported at rates of 3.7 to 10.9% in several studies [7, 9, 17, 20]. Our investigation revealed that postoperative inflammation was higher in eyes in which iris retractors had been used. Consistent with our results, Abbouda et al. suggested that the use of iris retractors induced postoperative inflammation [7]. Belovay et al. also noted that excessive surgical manipulations and structural damage could trigger severe postoperative inflammation [21]. However, no significant relation was found between the release of synechiae and postoperative inflammation in our study. The level of postoperative anterior chamber inflammation was significantly lower in hypochromic eyes of FUS patients. This lower inflammation level may be a consequence of decreased release of proinflammatory cytokines due to iris atrophy. Fang et al. reported a higher postoperative inflammation level in eyes with recurrent uveitis [22]. We did not observe this relationship between etiological causes and postoperative inflammation levels. Hydrophilic IOLs were suggested to be more biocompatible and to cause less postoperative inflammation, but we found no significant relationship between the implanted IOL type and the postoperative inflammation level [23].

### Recurrence

Liu et al. reported a recurrence rate of 40.3% in their study of 226 eyes at the end of their 16-month follow-up [5]. Estafanous et al. reported a mean 19-month remission before surgery and a recurrence rate of 41% at the end of their 20-month follow-up [9]. In our study, at least one recurrence was observed in 55.7% of all eyes, which was a higher rate than was reported in previous studies. Therefore, the high recurrence rate seen in our study may be associated with high incidences of some etiological causes, such as BU and presumed herpetic uveitis, which are known to recur frequently. Consistent with our proposal, Kawaguchi et al. emphasized the relation between uveitis etiology and recurrence rate [24]. In addition, Matsuo et al. noted higher recurrence rates in eyes with a preoperative remission period of less than 1 year [25]. Our findings showed that the postoperative recurrence risk was 2.3-fold higher in eyes that had a preoperative remission period of less than 1 year. As emphasized in previous studies, the remission period had a great impact on postoperative recurrence; however, no consensus exists to date regarding the preoperative remission period [26–28].

### Posterior capsule Opacification

Kosker et al. reported PCO development in 12.7% of all eyes during a six-month follow-up [17]. Estafanous et al. announced a rate of PCO development of 62% and noted that PCO occurred more frequently with longer follow-up [9]. The high degree of PCO development in our study may be the result of longer follow-up, since we recorded that PCO development was positively correlated with the duration of the follow-up period. Kosker et al. suggested that there was a higher risk of PCO development in eyes with postoperative anterior chamber reaction [17]. However, no such relation was observed in our study. We found that eyes with earlier recurrence showed earlier PCO development and that eyes with a higher number of recurrences were more prone to developing PCO. Chronic inflammation plays a part in PCO development, and cataract surgery triggers inflammatory mediators that have been shown to promote lens epithelial cell proliferation and migration [29]. In addition to surgical stress, underlying chronic uveitis as well can cause elevated inflammatory mediator levels that promote PCO development in the long-term [30]. PCO development also shows a dependence on the type of implanted IOL [6, 7]. Alio et al. and Abela-Formanek et al. emphasized that PCO development was significantly more frequent in eyes implanted with hydrophilic IOLs [6, 23]. In our study, the PCO development risk was 2.8-fold higher in eyes implanted with hydrophilic IOLs than in those that received hydrophobic IOLs. The lower PCO rate in eyes with hydrophobic IOLs may be related to a tight capsule and IOL opposition that prevent lens epithelial cell migration between the lens and IOL [31].

### Cystoid macular edema

In our study, 16% of all eyes developed CME during follow-up. Our results showed that the eyes with earlier postoperative recurrence showed earlier CME development. Consistent with our results, Liu et al. and Estafanous et al. found that CME occurred more commonly in eyes with more recurrence during 16-month and 20-month follow-up periods, respectively [5, 9]. Elgohary et al. noted that the risk of CME development was higher in eyes that demonstrated recurrence in the first 3 months after surgery [18]. Agrawal et al. shared that a preoperative history of CME increased the risk of development of postoperative CME [13]. Consistently with their findings, our investigation revealed that the risk of CME development after phacoemulsification was 6.7-fold higher in eyes with a history of previous CME than in those with no CME history, and this higher risk may be the consequence of the additive effect of surgical stress and the ongoing uveitic process. This is because the underlying uveitic process and surgical stress trigger the secretion of inflammatory mediators and damage the integrity of the blood–retina barrier and pump function of the retinal pigment epithelium, causing fluid accumulation [32]. CME has great negative impact on visual acuity, if it left untreated [33]. Management of postoperative and uveitic CME rely on the anti-inflammatory agents such as non-steroid anti-inflammatory drugs and corticosteroids; furthermore, immunomodulatory drugs may be required [33, 34]. In the present study, we used topical non-steroid anti-inflammatory drugs and topical corticosteroids in combination for the initial treatment of CME. In the case of refractory CME, we used periocular or intravitreal corticosteroids as alternative therapy.

### Intraocular pressure and Glaucoma

In our study, 8.5% of all eyes required additional antiglaucomatous treatment due to the elevated IOP after phacoemulsification. Previously reported postoperative elevated IOP rates vary between 4.6 and 28.9% [4, 7, 20, 24]. IOP control may be difficult to maintain in eyes with uveitis after phacoemulsification [5, 7, 35]. Postoperatively increased IOP may be related to release of inflammatory mediators, residual viscoelastic device, pigment dispersion, and retained lens material [36]. Beside surgical factors, ongoing inflammatory processes and corticosteroid treatment may also occlude drainage ways and increase IOP in patients with uveitis [37, 38]. Our study revealed that postoperative glaucoma development was directly correlated with postoperative high IOP values. Eyes with a higher postoperative IOP were found to be 5.4-fold more prone to developing glaucoma.

In literature, it has been reported that the risk of vision loss was higher in pseudophakic uveitic eyes compared to phakic uveitic eyes [39]. However, it should be also considered that higher vision loss risk in pseudophakic uveitic eyes may be attributed to the likelihood of cataract development in patients with more severe course of uveitis with frequent complications [39]. In a meta-analysis, it was suggested that majority of uveitic cataract cases reach normal or near-normal visual acuity after cataract surgery but not as frequent as non-uveitic ager related cataract cases [10]. Several studies have also noted that eyes with well-controlled uveitis had favorable outcomes [3, 5, 13, 40].

The main limitation of our study was that it was conducted at a tertiary referral specialized eye center, so our study population did not represent the normal population and includes patients with refractory uveitis. This might have caused bias in our results. In addition, some of cases with a favorable postoperative course and good prognosis were lost to follow-up prematurely and this could have inadvertently distorted our results. Also, we could not evaluate grade of cataract, which may affect long-term outcomes.

## Conclusion

Our study showed that phacoemulsification and IOL implantation in patients with uveitis yield desirable visual outcomes and revealed the risk factors associated with low visual acuity in long-term follow-up. Low CDVA outcomes were associated with complications of uveitis itself rather than with cataract surgery and its complications.

## Data Availability

The datasets used and/or analyzed during the current study are available from the corresponding author on reasonable request.

## Abbreviations

BU: Behçet uveitis
CDVA: Corrected distance visual acuity
CI: Confidence interval
CME: Cystoid macular edema
FUS: Fuchs uveitis syndrome
IOL: Intraocular pressure
IOL: Intraocular lens
PCO: Posterior capsule opacification
RR: Relative risk
